# New Mutations in Chronic Lymphocytic Leukemia Identified by Target Enrichment and Deep Sequencing

**DOI:** 10.1371/journal.pone.0038158

**Published:** 2012-06-01

**Authors:** Elena Doménech, Gonzalo Gómez-López, Daniel Gzlez-Peña, Mar López, Beatriz Herreros, Juliane Menezes, Natalia Gómez-Lozano, Angel Carro, Osvaldo Graña, David G. Pisano, Orlando Domínguez, José A. García-Marco, Miguel A. Piris, Margarita Sánchez-Beato

**Affiliations:** 1 Molecular Pathology Programme, Spanish National Cancer Research Centre (CNIO), Madrid, Spain; 2 Structural Biology and Biocomputing Programme, Spanish National Cancer Research Centre (CNIO), Madrid, Spain; 3 University of Vigo, Pontevedra, Spain; 4 Hospital Universitario Puerta de Hierro-Majadahonda, Madrid, Spain; 5 Biotechnology Programme, Spanish National Cancer Research Centre (CNIO), Madrid, Spain; 6 IFIMAV, Fundación Marqués de Valdecilla, Santander, Spain; Hemocentro de Ribeirão Preto, HC-FMRP-USP, Brazil

## Abstract

Chronic lymphocytic leukemia (CLL) is a heterogeneous disease without a well-defined genetic alteration responsible for the onset of the disease. Several lines of evidence coincide in identifying stimulatory and growth signals delivered by B-cell receptor (BCR), and co-receptors together with NFkB pathway, as being the driving force in B-cell survival in CLL. However, the molecular mechanism responsible for this activation has not been identified. Based on the hypothesis that BCR activation may depend on somatic mutations of the BCR and related pathways we have performed a complete mutational screening of 301 selected genes associated with BCR signaling and related pathways using massive parallel sequencing technology in 10 CLL cases. Four mutated genes in coding regions (*KRAS*, *SMARCA2*, *NFKBIE* and *PRKD3*) have been confirmed by capillary sequencing. In conclusion, this study identifies new genes mutated in CLL, all of them in cases with progressive disease, and demonstrates that next-generation sequencing technologies applied to selected genes or pathways of interest are powerful tools for identifying novel mutational changes.

## Introduction

Chronic lymphocytic leukemia/small lymphocytic lymphoma (CLL/SLL) is the most common adult leukemia in the western world. Most CLL cells are inert and arrested in G0/G1 of the cell cycle. However, it is the progressive accumulation of tumor cells that ultimately leads to symptomatic disease. Pathogenic mechanisms involve multiple external events (for instance, microenvironmental and antigenic stimuli) and internal events (genetic and epigenetic) [1] that are associated with the transformation, progression and evolution of CLL.

CLL is a heterogeneous disease. Some patients progress rapidly and have short survival, whereas others have a more stable clinical course that may not need treatment for years.

Previous studies have demonstrated that overexpression of genes belonging to the B-cell receptor (BCR) pathway, and regulators, such as CD79B, BTK, TNFRSF7 (CD27), IKB2 and SYK, are correlated with a more aggressive behavior in CLL [2], [3], [4], [5]. Along with this finding, oncogenic mutations in multiple genes such as CARD11 [6], LYN [7] and A20 [8], among others, are described as causing deregulation in the BCR and related pathways, such as NFkB in B-cell lymphoma.

Several lines of evidence coincide in revealing that stimulatory and growth signals delivered by BCR and co-receptors enable lymphoma cells to avoid apoptosis and so to proliferate, thereby constituting the driving force for B-cell survival. These seem to be common features of lymphoma pathogenesis. It has been proved that maintained signaling mediated via this pathway is an essential mechanism for tumor cell survival in some lymphoma types, such as CLL and diffuse large B-cell lymphoma (DLBCL) [9], [10], [11]. However, the molecular mechanism responsible for the activation has not been fully clarified. On the basis of the hypothesis that BCR activation may depend on somatic mutations of the BCR genes and related pathways [12], we have used deep sequencing technology to investigate the existence of changes in the coding and regulatory sequences of a selection of 301 genes that are associated with BCR, or related to signaling from the B-cell receptor, such as the NFKB and JAK/STAT pathways.

## Results

Clinical data of the 10 patients included in the study are shown in Table 1. It is a heterogeneous group of patients in terms of staging, genetic alterations and prognostic. Four of the patients showed progressive diseases, which is defined as the need for therapy, while stable disease is defined as those cases that do not require therapy.

**Table 1:** Clinical features, evolution and biological characteristics of the ten CLL patients studied.

Samples	Follow-up (months)	Rai/Binet at diagnosis	Rai/Binet current stage	Cytogenetic alterations	Lymphocytes (µl)	CD19/CD5 (%)	ZAP-70	CD38 (%)	Rearrangement	IGHV homology	β2 microglobulin	LDH
CLL1	102	A/0	A/I	Trisomy 12	85,000	92	POSITIVE	12.5	IGVH1-2	100	1.4	417
CLL2	12	A/0	A/0 progressive	Rearrangement IgH	180,000	90	POSITIVE	14.4	IGHV3-11	99.6	12.5*	403
CLL3	108	A/I	A/I	del13q	55,000	80	NEGATIVE	3.9	IGHV1-2	94.3	1.4	325
CLL4	134	A/0	A/I	del13q	40,000	90	NEGATIVE	0.2	IGHV2-5	96.4	1.6	315
CLL5	1.5	B/II	B/II progressive	del17p13, c-myc amplification	170,000	94	POSITIVE	15.4	IGHV2-70	99.5	5.1	1044
CLL6	9	B/II	B/II	del13q	17,400	80	POSITIVE	0	IGHV3-21	98.6	1.7	523
CLL7	16	B/II	B/II progressive	Trisomy 12	53,000	93	POSITIVE	13	IGHV1-69	99.2	1.8	491
CLL8	34	A/0	A/0	del13q, del11q	10,300	62	NEGATIVE	5.4	DH	ND	2.1	321
CLL9	108	A/0	C/III progressive	del13q	171,000	96	NEGATIVE	0.1	IGHV3-21	92.8	2.2	572
CLL10	60	A/I	A/I	del13q	22,000	82	NEGATIVE	4.5	IGHV1-69	100	1.4	423

Summary of the prognosis factors considered in the clinic and genetic lesions that the ten patients harbor. ND = not done, del = deletion.

FISH analysis revealed a variety of cytogenetic abnormalities, including del(13q), del(17p), del(11q) and trisomy 12. The most frequent of these was del(13q), which was found in 6 of the 10 samples.

To explore whether BCR activation depends on somatic mutations in BCR-signaling genes and related pathways, we used single-end deep sequencing to perform a mutational screening of the exonic and regulatory regions of 301 selected genes (Table S1). The study pipeline and DNA sequencing data, including QC metrics, are summarized in Figure S1 and Table S2.

**Figure 1:**
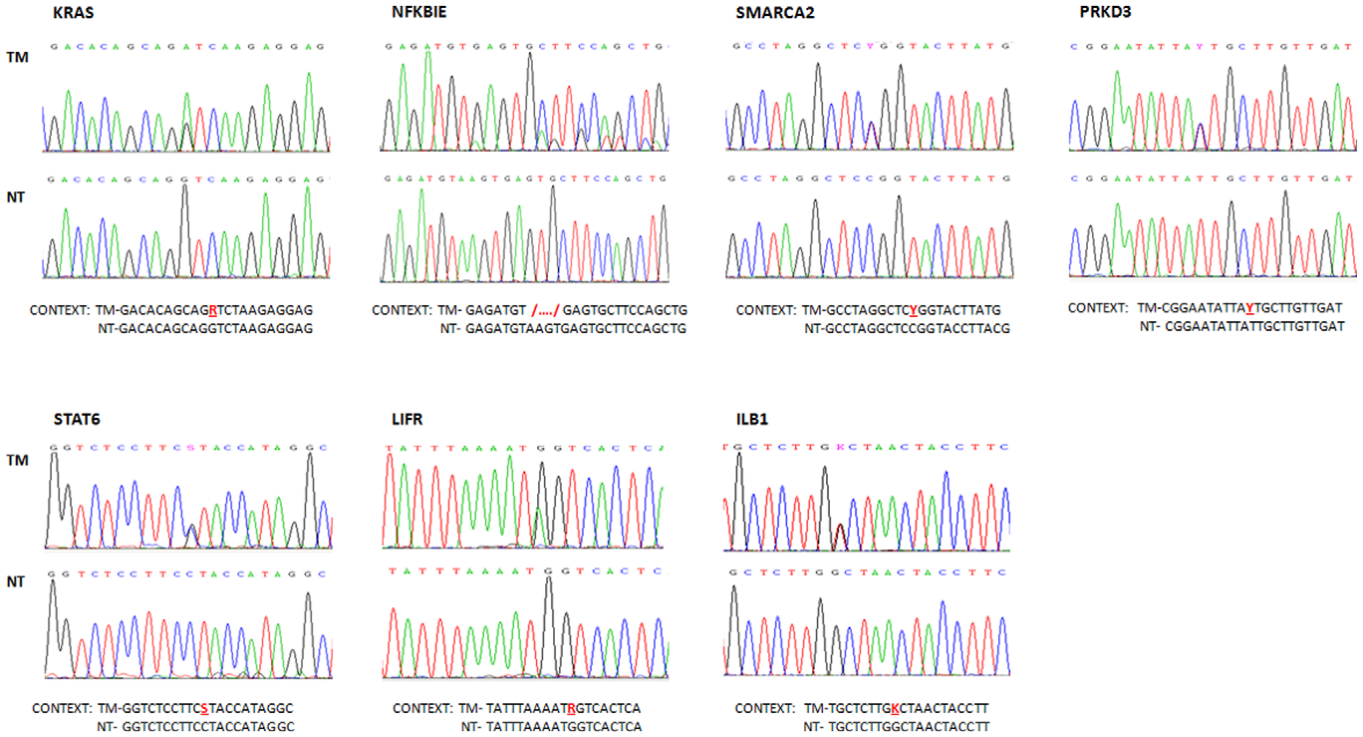
Mutation validation. Capillary sequencing chromatograms confirming the seven putative variants. *KRAS*, *SMARCA2*, *PRKD3*, *STAT6*, *LIFR* and *ILB1* showed one point mutation each, depicted as two peaks in the tumoral DNA chromatogram. For *NFKBIE*, the 4-bp deletion was also confirmed. NT = non-tumoral, TM = tumoral. R = A or G; Y = C or T; S = C or G; K = T or G; /…./ = 4-bp deletion.

In summary, detected variants were ranked by their VCF QUAL parameter and manually reviewed in the IGV browser (http://www.broadinstitute.org/igv/). The percentage of reads supporting the mutation of the total number of reads at a given position was taken as >20% with a minimum depth of 30× in tumor DNA, and <0.5% in normal DNA [13]. Selected candidates (non-synonymous, nonsense or frameshift mutations, or that affecting intronic or UTR regions) were validated by PCR amplification of the genomic region and capillary sequencing (File S1). Seven variants were selected for validation, and all of them were validated by capillary sequencing (Table 2 and Figure 1).

**Table 2:** Validated somatic mutation.

Samples	Position (hg19)	Description	Substitution	Region	SNV Type	Prediction	Gene Name
CLL2	chr5: 38478732	G-a	NA	3′UTR	Non-coding	NS	LIFR
CLL5	chr6:44232738	-GTAA	Frame-shift (253–254)	Exon CDS	Non-synonymous	Truncated	NFKBIE
CLL5	chr2:113590942	G-t	NA	Intronic	Non-coding	NS	IL1B
CLL7	chr12:57493232	G-c	NA	Intronic	Non-coding	NS	STAT6
CLL7	chr12:25380279	GGT-GaT	G60D	Exon CDS	Non-synonymous	Damaging	KRAS
CLL7	chr9:2182166	CGG-CaG	R1462Q	Exon CDS	Non-synonymous	Damaging	SMARCA2
CLL9	chr2:37505118	AAT-AgT	N396S	Exon CDS	Non-synonymous	Tolerated	PRKD3

CDS: Coding sequence. NA: not annotated. Chr: chromosome.

**Figure 2:**
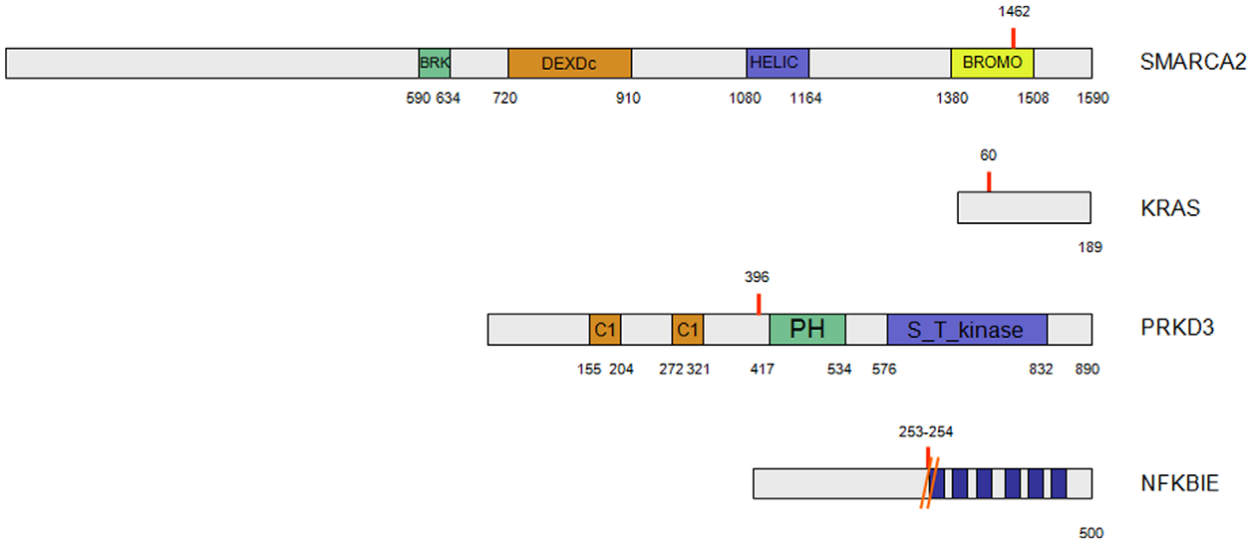
Schematic representation of the mutated proteins with the primary structural domains highlighted.

Three of these mutations (present in *KRAS*, *SMARCA2* and *PRKD3* genes) were non-synonymous point mutations, located in the coding region of the genes. Two were G>A transitions and one was an A>G change. There was also a frame-shift mutation due to a 4-bp deletion (in *NFKBIE* gene) that resulted in a stop codon 13 aminoacids downstream from it, which would generate a truncated protein. The Ensembl Variant Effect Predictor (VEP) [14] predicted that mutations in two of the genes (*KRAS* and *SMARCA2*) were deleterious to protein function (Figure 2 and Table 2).

The mutation in SMARCA2 lies inside the bromodomain of the protein. Using the NMR structure of the bromodomain (PDB code 2DAT, chain A) we were able to check that the WT residue is placed in one of the loops of the bromodomain, opposite to the region of the helical bundle involved in the binding with the acetyl-lisine, and so the effect of this mutation is difficult to predict (data not shown).

Finally, there were two point mutations located at intronic sites (*STAT6* and *ILB1*) and one in the 3′ UTR (*LIFR*).

Mutations in coding sequences were detected in the 3 cases with a progressive course. CLL7 and CLL5 (with 2 and 1 different mutations, respectively) exhibited adverse prognosis factors, such as unmutated IGVH (IGHV1-69 and IGHV2-70, respectively) and ZAP-70 positivity; case CLL5 also harbor 17p13 deletion and c-myc amplification. Case CLL9 progressed from stage RAI-BINET A/0 at diagnosis to C/III. This case harbors del(13q) and mutated IGVH3-21, a finding usually associated with a poor prognosis, irrespective of the IGVH mutational status [15] (Table 1).

## Discussion

Based on the hypothesis that BCR activation may depend on somatic mutations of the BCR and related pathways we have performed a complete mutational screening of 301 selected genes associated with BCR signaling and related pathways, such as NFkB, using massive parallel sequencing technology in 10 CLL cases. Three out of four progressive cases included in the study have mutated genes in the coding region. The mutated genes were *KRAS*, *SMARCA2*, *NFKBIE and PRKD3*, all of which play a role in BCR, NFkB and related signaling pathways.

The KRAS (v-Ki-ras2 Kirsten rat sarcoma viral oncogene homolog) protein is a GDP/GTP-binding protein that acts as an intracellular signal transducer, a molecular regulator in a variety of signal transduction pathways, such as BCR signaling pathways (Figure S2), or controlling phenomena such as cytoskeleton integrity, proliferation differentiation, adhesion, cell migration and apoptosis. From 17 to 25% of all human tumors have the activating *KRAS* mutation, but it has been described in the hematopoietic and lymphoid tissue of only about 5% of cases (COSMIC database; http://www.sanger.ac.uk/genetics/CGP/cosmic/). Specifically in CLL, *KRAS* mutations have been described only exceptionally [16], [17]. The mutation identified here, located at codon 60, has not been described before, but is located in the hotspot region of codons 59, 61 and 62. Therefore, the putative effect of this mutation on protein function should be similar, leading to the activation of the protein.

NFKBIE (nuclear factor of kappa light polypeptide gene enhancer in B-cell inhibitor epsilon) is upregulated following NF-κB activation. NFKBIE is able to inhibit NF-κB-directed transactivation via cytoplasmic retention of REL proteins. For *NFKBIE*, inactivating mutations have been described in Hodgkin/Reed-Sternberg cells [18]. In the paper by Quesada et al.[19] a non-sense mutation in a CLL case is also described (chr6: 44230329; C/A) and in the COSMIC database, two mutations are described in breast cancer, one of which is a nonsense mutation, like the one described here. This kind of mutation should give rise to a truncated protein without the ankyrin motif mediating the protein–protein interaction and would eventually be degraded. The inactivation of NFKBIE by mutation would lead to the sustained activation of the NFKB pathway (Figure S2), which has previously been linked to CLL pathogenesis [20]

SMARCA2 (SWI/SNF related, matrix associated, actin dependent regulator of chromatin, subfamily a, member 2), also known as SNF2L2 or BRM, is part of the large ATP-dependent chromatin remodeling complex SWI/SNF, which is required for transcriptional activation of genes normally repressed by chromatin. It has been described as a mediator of NFkB transcriptional activation, and is also associated with BCR signaling mediated by BTK [21], [22]. (Figure S2). Four substitution mutations are reported for the *SMARCA2* gene in the COSMIC database and in the ICGC portal (http://dcc.icgc.org/); two mutations were identified and validated in CLL (intronic) and glioblastoma (non-synonymous coding). The mutation found in the present study is located in the bromodomain, whose function is not completely clear although it may play a role in the assembly or activity of multi-component complexes involved in transcriptional activation. Mutations have been described in other members of the same family, such as *ARID1A* in around 50% of ovarian clear cell carcinomas and in gastric cancer [13], [23], [24], and *SMARCA4/BRG1* in lung tumors [25], [26]. In both cases, mutations are located throughout the entire gene coding sequence.

PRKD3 (protein kinase D3) belongs to the protein kinase C family of serine- and threonine-specific protein kinases that can be activated after BCR engagement [27] by calcium and the second messenger, diacylglycerol (Figure S2). It phosphorylates a wide variety of protein targets and is involved in diverse cellular signaling pathways [28]. Only sporadic mutations have been described in the *PRKD3* gene in lung adenocarcinoma, ovary and brain glioma (COSMIC and ICGC databases).

The other mutations found in *STAT6*, *IL1B* and *LIFR*, are located in intronic regions and their consequences are therefore not clear.

Recently, several groups have identified mutations in CLL using exome sequencing [19], , *NOTCH1* being the most frequently mutated gene that behaves as an independent predictor of poor survival [30]. Other mutated genes identified in these studies were *SF3B1* [19], [31], *MYD88*, *KLHL6* and *XPO1* [29], *TP53*, *PLEKHG5*, *TGM7* and *BIRC3* [30].

Given the small number of patients included in the series, this study is unable to conclude statistical significance. To infer a possible role and relevance for each mutated gene described, they need to be validated in a larger series of patients and through functional studies. Nevertheless, the findings are consistent with the idea that, at least partially, BCR and NFkB signaling and related pathways in CLL cells could depend on somatic mutations.

In conclusion, this study identifies new genes mutated in CLL and demonstrates that next-generation sequencing technologies applied to selected genes or pathways of interest are powerful tools for identifying novel mutational changes.

## Materials and Methods

### 1. Clinical samples

The current study was approved by the institutional Ethics Committee of the “Instituto de Salud Carlos III” (CEI PI 03_2011). Fresh blood samples from 10 untreated CLL patients were obtained from Hospital Universitario Puerta de Hierro-Majadahonda, with the collaboration of the CNIO Tumour Bank, in adherence with the protocols of the Ethics Committee of the Instituto de Salud Carlos III. All patients involved gave written informed consent for their participation in the study. Clinical data were collected according to the criteria of the National Cancer Institute (NCI) Working Group [32].

Tumoral DNA was collected from peripheral blood cells, CD19+ B-cell, then purified using the RossetteSep Human B cell enrichment kit (StemCell Technologies) or an immunomagnetic purification process (Myltenyi Biotec). Tumor cell purity was taken to be the ratio CD19/CD3, which was measured by FACS. The tumor B-cell purity of CLL samples after purification ranged from 88.00 to 99.80%. Non-tumoral DNA from each patient was collected from oral mucosa samples.

DNA was extracted by the phenol–chloroform method and the quality and quantity of purified DNA was assessed by fluorometry (Qubit, Invitrogen) and gel electrophoresis.

### 2. Target enrichment and sequencing

#### Gene selection and bait design

To select genes we browsed the KEGG and BioCarta databases and previously published data on CLL and genes acting on BCR signaling and related pathways, such as NFkB and JAK/STAT [2],[3],[11],[12],[20] (Table S1).

The USCS Genome Browser (http://genome.ucsc.edu/) was used to select exonic sequences. A region 200 bp downstream of the start transcription point was also included. The coordinates of the genomic sequences were based on UCSC hg19.

The web-based design tool eArray (Agilent Technologies Inc, Santa Clara, CA, USA) was used to design the baits for the SureSelect Target Enrichment System kit.

Biotinylated-RNA 120-mer capture probes had sequences complementary to those of the selected regions, and extended 20 bp upstream and downstream of each region. Repeat masked regions were avoided in the design. The targeted region was 1.36 Mbp (up to 0.04% of the human genome), thereby consisting of 4,000 exons and 580 ds regions. 35,985 capture-centered probes were designed, allowing an overlap of 60 bp and a 4× tiling frequency. The average tiling frequency achieved was 3.2×, because exons shorter than 120 bp had a unique complementary probe.

Selected sequences were enriched for each library using the SureSelect custom design Kit (Agilent Technologies).

#### Library preparation and sequencing

The protocol for “SureSelect Target Enrichment System” kit (Agilent Technologies) combined with the “Genomic DNA Sample Prep for single-end sequencing” guide (Illumina, San Diego, CA, USA) were used for sample sequence enrichment (31) and library preparation. Briefly, 1–3 µg of high molecular weight genomic DNA from each sample was fragmented by acoustic shearing on a Covaris S2 instrument. Fractions of 150–300 bp were ligated to Illumina's adapters and PCR-amplified for 6 cycles. Exon Enrichment: 300 ng of whole library were hybridized to SureSelect Oligo Capture kit for 24 h at 65°C. Biotinylated hybrids were captured, and the enriched libraries were completed with 12 cycles of PCR. The resulting purified DNA library was applied to an Illumina flow cell for cluster generation and sequenced using the Illumina Genome Analyzer IIx for 36 bases in a single-read format by following the manufacturer's protocols.

### 3. Data analysis

#### Alignment, mapping and identification of somatic variants

Sequencing data were first checked by FastQC (http://www.bioinformatics.bbsrc.ac.uk/projects/fastqc/) for quality control checks on raw sequence data and then aligned to the human reference genome (GRCh37) using Burrows-Wheeler alignment (BWA). That reads unmapped by BWA [33] were realigned using BFAST [34]. Somatic variants were identified using the Unified Genotyper v2 available at the GATK [35]. For variant calling we used GATK Unified Genotyper v2 applying the “Discovery” genotyping mode and default parameters for filtering. Thus, SNPs available at dbSNP 132 (hg19) and those reported by the 1000 Genomes Project were filtered out from VCF output files. The GATK QUAL field was employed for ranking selected somatic variants. The percentage of reads supporting the mutation of the total number of reads at a given position was taken as >20% in the tumor DNA, and <0.5% in normal DNA [13]. Only those variants showing a depth 30× in tumoral samples, were considered for validation. Data has been deposited in the Sequence Read Archive (SRA) database (http://www.ncbi.nlm.nih.gov/sra) (SRA049097).

Biological impact predictions for detected variants were obtained from Ensembl Variant Effect Predictor (VEP: http://www.ensembl.org/tools.html) [14].

## Supporting Information

Figure S1 **Workflow.** The USCS Genome Browser (http://genome.ucsc.edu/) was used to select exonic sequences and the web-based design tool eArray (Agilent Technologies) was used to design the baits for the SureSelect Target Enrichment System kit. The library was prepared and sequenced in a GA2 sequencer from Illumina. Sequencing data were aligned to the human reference genome (GRCh37) using Burrows-Wheeler alignment (BWA) and BFAST. Somatic variants were identified using the Unified Genotyper v2 available at the GATK. Somatic variants were filtered by i) known SNPs available at dbSNP 132 (hg19) and those reported by 1000 Genome Project, ii) changes present in the matched germinal DNA and iii) synonymous changes. Ensemble VEP was used to predict biological effect and, finally the variants were ranked by GATK quality score, manually reviewed and validated by capillary sequencing.
(PPT)

Figure S2 **Mutations in components of B-cell receptor signaling pathways.** A modified KEGG (http://www.genome.jp/kegg/) pathway including B-cell receptor signaling is depicted. Somatically mutated genes in CLL are highlighted in red.
(PPT)

File S1 **Supplementary Materials and Methods: Validation and sequencing.**
(DOC)

Table S1 **List of sequenced genes.** 301 genes, belonging to the BCR, NFkB and JAK/STAT signaling pathways, were selected for sequencing.
(XLS)

Table S2 **Quality control.** Quality metrics of alignment, mapping and coverage depth from the two independent experiments. Mbp: Megabase pair. T: Tumoral. NT: Non-tumoral.
(XLS)
