# Supplementary material for: New Mutations in Chronic Lymphocytic Leukemia Identified by Target Enrichment and Deep Sequencing
Source: PLoS One. 2012 Jun 1;7(6):e38158. doi: 10.1371/journal.pone.0038158 (PMC3365884; doi:10.1371/journal.pone.0038158)
Supplement: Figure S1 — Workflow. The USCS Genome Browser (http://genome.ucsc.edu/) was used to select exonic sequences and the web-based design tool eArray (Agilent Technologies) was used to design the baits for the SureSelect Target Enrichment System kit. The library was prepared and sequenced in a GA2 sequencer from Illumina. Sequencing data were aligned to the human reference genome (GRCh37) using Burrows-Wheeler alignment (BWA) and BFAST. Somatic variants were identified using the Unified Genotyper v2 available at the GATK. Somatic variants were filtered by i) known SNPs available at dbSNP 132 (hg19) and those reported by 1000 Genome Project, ii) changes present in the matched germinal DNA and iii) synonymous changes. Ensemble VEP was used to predict biological effect and, finally the variants were ranked by GATK quality score, manually reviewed and validated by capillary sequencing. (PPT) [file pone.0038158.s001.ppt]

## Slide 1
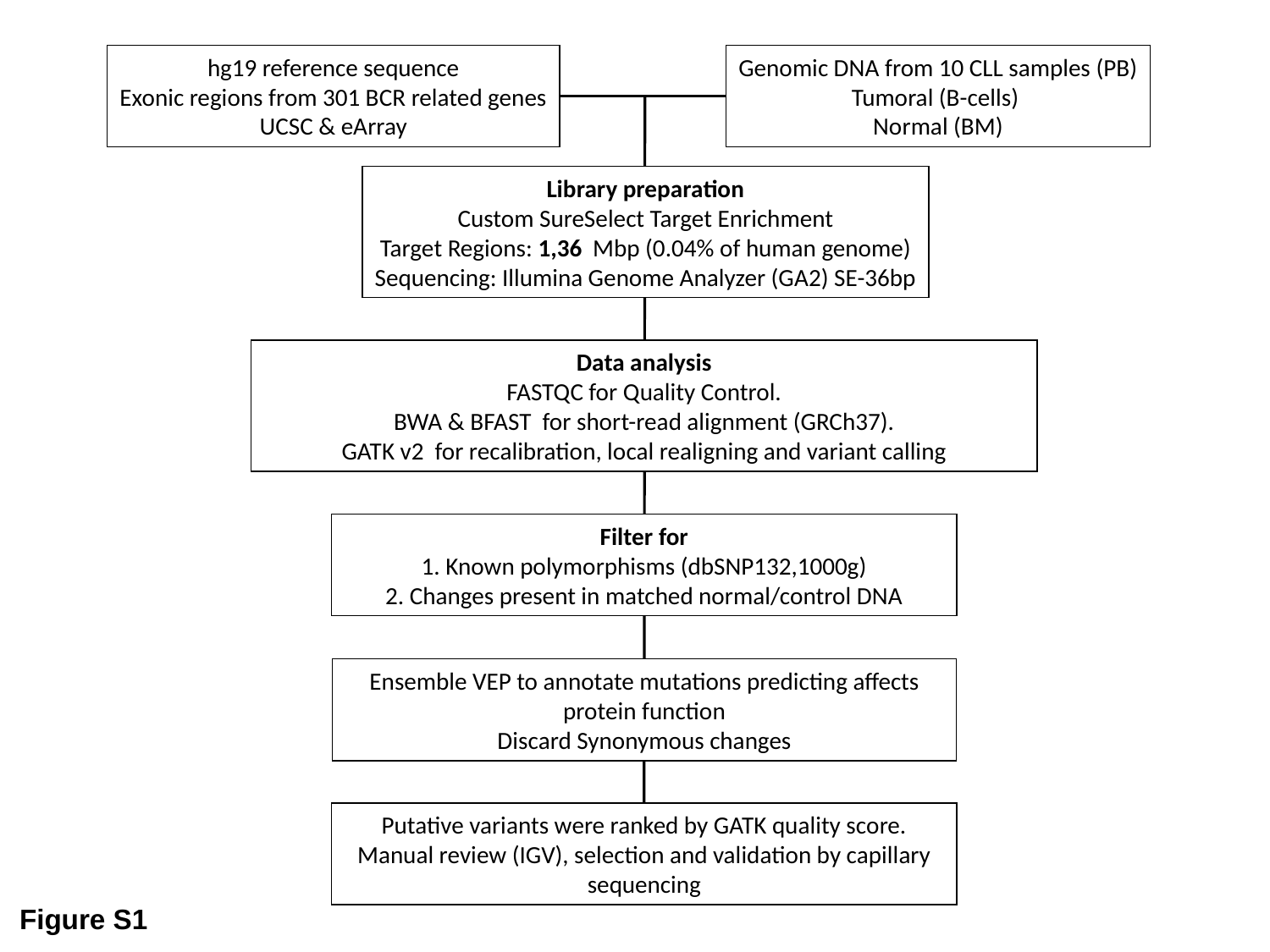

hg19 reference sequence
Exonic regions from 301 BCR related genes
UCSC & eArray
Genomic DNA from 10 CLL samples (PB)
Tumoral (B-cells)
Normal (BM)
Library preparation
Custom SureSelect Target Enrichment
Target Regions: 1,36 Mbp (0.04% of human genome)
Sequencing: Illumina Genome Analyzer (GA2) SE-36bp
Data analysis
FASTQC for Quality Control.
BWA & BFAST for short-read alignment (GRCh37).
GATK v2 for recalibration, local realigning and variant calling
Filter for
1. Known polymorphisms (dbSNP132,1000g)
2. Changes present in matched normal/control DNA
Ensemble VEP to annotate mutations predicting affects protein function
Discard Synonymous changes
Putative variants were ranked by GATK quality score. Manual review (IGV), selection and validation by capillary sequencing
Figure S1
