# Supplementary material for: New Mutations in Chronic Lymphocytic Leukemia Identified by Target Enrichment and Deep Sequencing
Source: PLoS One. 2012 Jun 1;7(6):e38158. doi: 10.1371/journal.pone.0038158 (PMC3365884; doi:10.1371/journal.pone.0038158)
Supplement: Figure S2 — Mutations in components of B-cell receptor signaling pathways. A modified KEGG (http://www.genome.jp/kegg/) pathway including B-cell receptor signaling is depicted. Somatically mutated genes in CLL are highlighted in red. (PPT) [file pone.0038158.s002.ppt]

## Slide 1
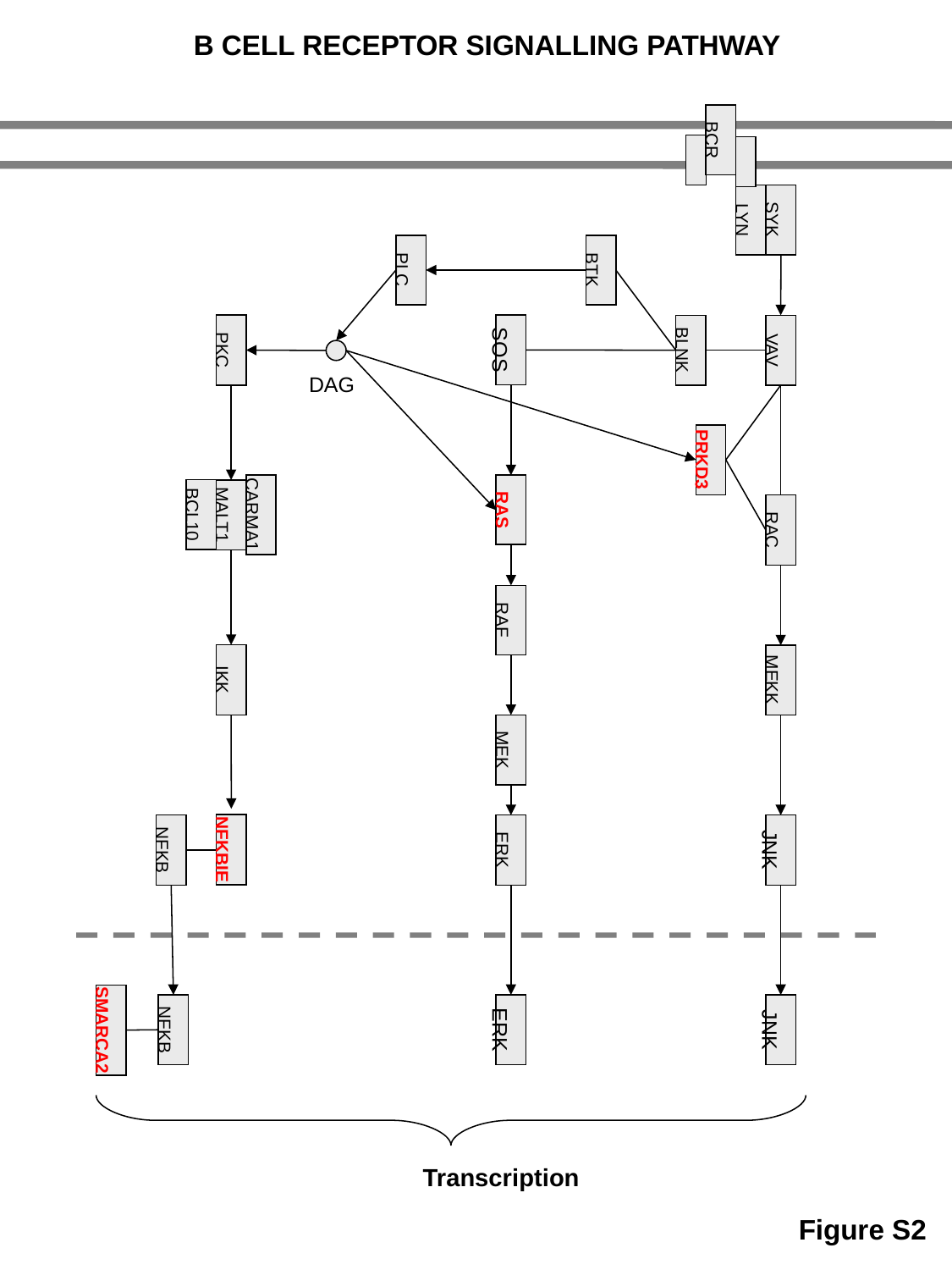

B CELL RECEPTOR SIGNALLING PATHWAY
BCR
LYN
SYK
PLC
BTK
PKC
SOS
BLNK
VAV
DAG
PRKD3
PRKD3
PRKD3
RAS
RAS
RAS
CARMA1
RAS
RAS
BCL10
MALT1
RAC
RAF
IKK
MEKK
MEK
NFKBIE
NFKBIE
NFKBIE
NFKBIE
IKB
NFKB
IKB
ERK
JNK
SMARCA2
SMARCA2
NFKB
ERK
JNK
Transcription
Figure S2
