# Supplementary material for: New Mutations in Chronic Lymphocytic Leukemia Identified by Target Enrichment and Deep Sequencing
Source: PLoS One. 2012 Jun 1;7(6):e38158. doi: 10.1371/journal.pone.0038158 (PMC3365884; doi:10.1371/journal.pone.0038158)
Supplement: File S1 — Supplementary Materials and Methods: Validation and sequencing. (DOC) [file pone.0038158.s003.doc]

**Supplementary Information.**

**Supplementary Materials and Methods**

**Validation and sequencing**

Primers for amplification and sequencing were designed using the primer 3 software (<http://frodo.wi.mit.edu/primer3>), checked with the USCS Genome Browser PCR *in silico* tool, and purchased from Sigma–Aldrich. Forward and reverse primer sequences 5´-3´, respectively,

for *PRKD3* are FW_CAGGTTATCCCTGCTGGTGT, RV_AAAAAGGCTTCATGTTCCTACAG;

for *NFKBIE* are FW_CGAATGGGGACTTGAAGGAT, RV_CTCGCTCACCTACACCCTGT;

for *KRAS* are FW_AAAGAAAGCCCTCCCCAGT, RV_TCAAGTCCTTTGCCCATTTT;

for *SMARCA2* are FW_GATCGCTGAATTTTCCTCTCC, RV_GAACAACGGGCACATTTACA;

for *STAT6* are FW_CCTTGGGCTTCTTGGGATAG, RV_CCCACCCTAGGTCCTCTCTC;

for ILB1 are FW_ TTGGTTTCCAGCCTTCTTTG, RV_ GGACAAGCTGAGGAAGATGC;

and for LIFR are FW_ GACCTAGATTGCATACATGTGCT, RV_AGTCAATTCACTTACAAGCCAGT.

Amplification was performed using DNA Polymerase HotStart (Finnzymes), and the reaction mix contained 1x final concentration of 10x HotStart Amplification Buffer (Finnzymes), 200 µM dNTPs (Invitrogen), 0.2 µM forward primer, 0.2 µM reverse primer, and 1.5 µM HotStart DNA polymerase in a final reaction volume of 25 µL. PCR conditions were: 5 min at 94 ˚C, 30 s at 94 ˚C, 30 s at 60 ˚C, 30 s at 72 ˚C (30 cycles), 10 min at 72 ˚C, ∞ at 10 ˚C. Agarose gel electrophoresis was performed with 2 µL of the reaction products to confirm primer specificity.

PCR products were then purified using QIAquick columns PCR Purification Kit (QIAGEN) or the exosap protocol and quantified using Nanodrop.

For the capillary sequencing reaction for each amplicon, about 6 ng/µL of DNA, and 5 pmol/µL of each forward and reverse primer were used.
